# On the Tincture of the Poppy, Made from the Entire Plant, as a Substitute for Laudanum

**Published:** 1821-12

**Authors:** Daniel Wilson


### *On the Tincture of the Poppy, made from the entire Plant, as a Substitute for Laudanum.* By Daniel Wilson, m.d.

IN reading the interesting remarks of Dr. Duncan, sen. of Edinburgh, on the preparation and use of the tincture of lettuce, I was led to suppose that a tincture of the papaver somniferum would prove a substitute for the tinctura opii, as well as for the celebrated gum from which that tincture is made. This idea presented itself on first reading Dr. Duncan's remarks about eighteen months since, and last summer I availed myself of an opportunity to subject my hypothesis to the test of experiment. Except the radix, I used every part of the plant, a quantity of which was obtained at the proper age for procuring the extract. In the eagerness of my pursuit, I dried the first parcel in the sun; but I was at once convinced that this would disappoint my views. A second parcel was procured, and carefully dried in the shade, and, having reduced it to a coarse powder, I proceeded with the intention of making a saturated tincture. The following is the result of my experiments:

℞. Pulv. pav. somnif. ℥ iv.

Sp. communis, ℥ xvj.

Mix, digest for eight days, and filter.

From these quantities I obtained eight or ten ounces of the tincture, which I forthwith introduced into my practice. Under the same principles which govern me in administering the tinctura opii, I gave the tinctura papaveri; and, in doses of double the quantity which I give of the former, I find the latter an effectual anodyne.

I am about trying a tincture made with the sp. vini, but my experiments are yet only in embryo. From tinct. pav. ℥ j. as above prepared, I procured extr. gr. xviij.; but, when formed into pills, they run together, unless given immediately, and are therefore fit only for extemporaneous use. I have laid aside the tinctura opii for some months past in my practice, not because I think it inferior to the tinc. papav. but because I believe the latter to be quite its equal as an anodyne; and, when considered in an economical point of view, its advantages are too obvious to require enumeration.

Professional pursuits, together with an unfortunate habit of procrastination, have prevented me from making this communication sooner. I hoped, too, that more time would have enabled me to make it more worthy of the attention of the faculty: but, as it is now uncertain whether I shall be able, for some months, to add to, or diversify, my experiments, I have resolved, imperfect as it is, to commit my project to the hands of others.

*Louisville; June 18th,* 1821.*

* The *Philadelphia Journal,* August 1821.

